# Unmet Healthcare Needs and Associated Factors Among Older Adults with Osteoporosis: A Cross-Sectional Analysis of the 2025 Korea Community Health Survey

**DOI:** 10.3390/healthcare14121670

**Published:** 2026-06-11

**Authors:** Eunjung Kim, Boyoung Kim, Hae Ran Kim

**Affiliations:** 1 Department of Nursing, Honam University, Gwangju 62399, Republic of Korea; 2 College of Nursing, Chonnam National University, Gwangju 61186, Republic of Korea; 3 Department of Nursing, College of Medicine, Chosun University, Gwangju 61452, Republic of Korea

**Keywords:** Andersen’s behavioral model, health literacy, older adults, osteoporosis, unmet healthcare needs

## Abstract

Background: Unmet healthcare needs are a significant public health concern among older adults, particularly those with chronic conditions such as osteoporosis. This study aimed to identify several key factors associated with unmet healthcare needs among older adults with osteoporosis using Andersen’s behavioral model. Methods: This observational, cross-sectional study examined data from the 2025 Korea Community Health Survey, analyzing responses from 20,988 individuals aged ≥ 65 years with physician-diagnosed osteoporosis. General characteristics were analyzed using descriptive statistics in terms of frequencies and percentages. Variables were categorized into predisposing, enabling, and need factors. Complex sample analyses were performed using the Rao–Scott chi-square test and multivariable logistic regression. Results: In the multivariable analysis, female sex (adjusted odds ratio [aOR] = 1.44, 95% confidence interval [CI]: 1.08–1.91), poor health literacy (aOR = 1.56, 95% CI: 1.31–1.87), and rural residence (aOR = 1.36, 95% CI: 1.18–1.58) were significantly associated with higher odds of unmet healthcare needs. Among the need factors, fall experience (aOR = 1.56, 95% CI: 1.34–1.83), pain or discomfort (aOR = 2.40, 95% CI: 1.93–2.99), and elevated stress (aOR = 2.37, 95% CI: 2.02–2.79) were also significantly associated with unmet healthcare needs. Conclusions: Beyond accessibility, unmet healthcare needs among older adults with osteoporosis in Korea were associated with cognitive, health-related, and regional factors. Interventions should prioritize improving health literacy, managing pain and psychological distress, and strengthening osteoporosis care pathways, specifically focusing on follow-up care and fragility fracture prevention.

## 1. Introduction

Osteoporosis is a chronic disease characterized by reduced bone mineral density and microarchitectural deterioration of bone tissue, resulting in increased susceptibility to fractures. This has become an increasingly significant public health concern globally, particularly in rapidly aging societies [1,2]. Korea is one such society where the prevalence of osteoporosis has risen, affecting approximately 23% of adults aged 50 years and older, and exhibiting substantially higher rates in women than in men (approximately 38% versus 7%) [3]. Furthermore, a Korean nationwide cohort study conducted between 2008 and 2018 reported an osteoporosis incidence of 18.4 cases per 1000 person-years among adults aged 50 years and older, highlighting this disease’s societal burden [4]. Although osteoporosis is often asymptomatic and difficult to detect in its early stages, the associated fragility fractures can lead to functional decline, diminished quality of life, increased mortality, and substantial socioeconomic burden. Consequently, continuous and timely management is essential [1,2,5,6]. Delayed diagnosis or suboptimal treatment can also contribute to the long-term burden of this disease, including the risk of future fractures, functional impairment, and a reduction in quality of life [2,7,8]. Further, delayed healthcare may interrupt essential steps in osteoporosis care, including diagnosis, risk assessment, medication adherence, fracture prevention, and rehabilitation after fracture [9]. This makes timely management key to avoiding such results, including access to specialized screening for the disease and its progression. This can encompass dual-energy X-ray absorptiometry (DXA) screening, pharmacologic treatments, fall-prevention programs, and follow-up care [9]. In older adults, mobility limitations, frailty, and other fracture-related disabilities may further restrict healthcare accessibility, making “unmet healthcare” needs particularly relevant in this population [7,10]. Unmet healthcare needs refer to the needs of individuals who are either unable or unmotivated to obtain necessary medical services despite recognizing their need for care. As an indicator, this is generally regarded as a measure of healthcare accessibility and inequality [11,12].

Prior research has demonstrated that these unmet needs are common among older adults and individuals with chronic diseases and are directly associated with health deterioration, functional limitation, and poorer quality of life [12,13,14]. Among individuals with chronic diseases requiring continuous management, unmet care is linked to increased risk of the disease progression and functional decline [13,14]. For patients with osteoporosis who require continuous management, unmet healthcare needs may pose additional challenges to effective disease management, representing a growing public health concern [1,15].

Andersen’s behavioral model provides a widely applied theoretical framework for understanding healthcare utilization and unmet healthcare needs [16]. This model suggests that healthcare utilization is shaped by three types of factors: predisposing factors, such as sociodemographic characteristics; enabling factors, such as resources and access to healthcare services; and need factors, such as health status and perceived or evaluated healthcare needs [16,17,18]. This model is particularly appropriate for examining unmet osteoporosis healthcare needs because healthcare access in this population may be influenced not only by clinical need but also by socioeconomic status, regional accessibility, transportation barriers, social support, and the ability to understand and use health information [16].

Previous research has identified sex, educational attainment, income, health status, and chronic conditions as significant determinants of unmet healthcare needs [19,20,21,22,23]. Other studies report key determinants such as healthcare accessibility, regional environment, and social support [22,24,25]. Notably, unmet care is particularly pronounced among individuals with limited financial resources or insufficient social support [11,26]. Older adults with osteoporosis may experience several of these barriers simultaneously. This suggests the need for a multidimensional framework that can account for individual characteristics, access-related concerns, and health-related needs among older adults with osteoporosis [16].

However, there is limited research on the unmet healthcare needs of patients with osteoporosis [1,14]. Previous studies have mainly addressed unmet healthcare needs in the general population or other chronic disease groups, leaving disease-specific evidence for osteoporosis insufficient [19]. In the case of Korea, few studies analyze recent nationally representative data on unmet healthcare needs among older Korean adults with osteoporosis. Additionally, few studies apply Andersen’s behavioral model to disease-specific populations such as those with osteoporosis [16].

Therefore, this study assesses the unmet healthcare needs among older adults with osteoporosis, using data from the Korea Community Health Survey (KCHS) conducted by the Korea Disease Control and Prevention Agency (KDCA), to identify the association with the factors within the framework of Andersen’s behavioral model. This approach provides us with a structured understanding of healthcare access barriers in this population, offering insights to inform public health strategies for long-term osteoporosis management and fragility fracture prevention.

## 2. Materials and Methods

### 2.1. Data Source and Study Participants

This study was designed as an observational, cross-sectional study and was reported in accordance with the Strengthening the Reporting of Observational Studies in Epidemiology (STROBE) guidelines. This study utilized data from the KCHS, a nationwide health survey conducted annually since 2008, encompassing adults aged 19 years and older. Participants were selected using a two-stage stratified cluster sampling design, and data were collected through computer-assisted personal interviewing (CAPI) conducted by trained interviewers [27]. All participants provided informed consent prior to enrollment.

For this study, we utilized data collected from the 2025 KCHS regarding health behaviors, healthcare utilization, and the social environment. From the total respondent pool, individuals aged 65 years or older were selected. Participants were classified as having osteoporosis if they responded “yes” to the question, “Have you ever been diagnosed with osteoporosis by a physician?” Unmet healthcare needs were defined based on the question, “During the past year, was there any time when you needed medical care (excluding dental care), such as examination or treatment, but did not receive it?” Respondents who answered “yes” were categorized as having unmet healthcare needs, whereas those who responded “no” were categorized as not having unmet healthcare needs. Out of the 21,466 participants aged 65 years and older with osteoporosis, 478 individuals were excluded due to missing values or reported “no need for medical care,” yielding a final sample of 20,988 participants.

### 2.2. Variables

We treated unmet healthcare needs as the dependent variable. Independent variables were selected from items included in the raw KCHS data based on the prior literature and categorized with reference to the KCHS guidelines [28]. Applying Andersen’s behavioral model, we classified these independent variables into predisposing, enabling, and need factors for our analysis [16].

Predisposing factors included sex, age, educational attainment, living with others, and health literacy. Sex was categorized as female or male. Age was grouped into 65–74 and ≥75 years. Educational attainment was categorized as ≥high school and ≤middle school based on the highest level attained. Living with others was defined based by household composition. Participants who reported a one-person household were classified as “no” (not living with others), while all other household types were classified as “yes” (living with others). Health literacy was evaluated using the question, “How difficult is it to find or obtain advice on health or medical information?” Responses of “very easy” or “somewhat easy” were classified as “good,” whereas responses of “somewhat difficult” or “very difficult” were classified as “poor.” Because the KCHS assesses this construct using a single item rather than a multidimensional health literacy scale, we treated this variable as a proxy indicator of perceived difficulty in obtaining health or medical information.

Enabling factors included household income, economic activity, and region. Household income was classified as high or low according to the median monthly household income of participants in the sample, with ≤1,600,000 KRW defined as “low” and >1,600,000 KRW as “high.” The median value enabled us to categorize all income groups within the study population. We did not use inflation-adjusted or region-adjusted income measures because these indicators were not available in the KCHS dataset. Economic activity was classified as employed or unemployed according to current employment status. Region was categorized as urban or rural based on administrative districts.

Needs factors comprised fall experience, pain/discomfort, and stress. Fall experience was categorized as “no” or “yes” based on whether the participant had experienced at least one fall within the past year. Pain/discomfort was categorized as “no” or “yes” based on whether the participant reported experiencing pain or discomfort in daily life. Stress was evaluated using the question, “How much stress do you usually feel in your daily life?” Responses of “a little” or “rarely” were categorized as low, whereas responses of “very much” or “a lot” were categorized as high.

### 2.3. Statistical Analyses

We captured the descriptive statistics as frequencies and percentages of the participants’ general characteristics. We conducted a Rao–Scott chi-square test to analyze the differences in the characteristics based on unmet healthcare needs. Subsequently, we performed univariable and multivariable logistic regression analyses to identify the factors associated with these unmet healthcare needs.

In the univariable analysis, we ran a simple logistic regression to assess the relationship between each independent variable and unmet healthcare needs. In the multivariable analysis, we included all the predisposing, enabling, and need factors simultaneously in the model. Our analyses accounted for the complex sampling design of the KCHS by incorporating stratification, clustering, and sampling weights.

We assessed multicollinearity among the independent variables using variance inflation factors (VIFs). The VIF values ranged from 1.03 to 1.33, indicating no evidence of problematic multicollinearity. Interaction terms were not included in the final model because the primary aim was to identify overall factors associated with unmet healthcare needs rather than to examine effect modification. Statistical analyses were performed using SAS version 9.4 (SAS Institute, Cary, NC, USA) with statistical significance set at *p* < 0.05.

### 2.4. Ethical Considerations

This study was exempt from ethical review as we utilized publicly available, de-identified data from the KCHS without the direct involvement of the participants. The original KCHS protocol received approval by the Institutional Review Board of the KDCA.

## 3. Results

Among the 20,988 older adults with osteoporosis, females accounted for 91.8% of the sample, and 57.6% were classified as having poor health literacy. Additionally, 24.6% reported experiencing a fall within the previous year, 64.9% reported pain or discomfort, and 19.4% reported high stress levels (Table 1).

Among these individuals with osteoporosis, 1126 (5.4%) reported unmet healthcare needs. The prevalence of unmet healthcare needs was significantly higher among females, individuals with poor health literacy, those with low income, and those residing in rural areas. It was also significantly higher among older adults aged ≥ 75 years, those with lower educational attainment, and those living alone (Table 2).

In the univariable analysis, sex, age, education attainment, health literacy, household income, region, fall experience, pain/discomfort, and stress were significantly associated with unmet healthcare needs. Specifically, the odds of unmet healthcare needs were highest among individuals with poor health literacy (odds ratio [OR] = 1.94, 95% confidence interval [CI]: 1.64–2.30), those reporting pain or discomfort (OR = 3.16, 95% CI: 2.56–3.91), and those experiencing high stress (OR = 2.85, 95% CI: 2.43–3.33) (Table 3).

In the multivariable logistic regression analysis, females (adjusted OR [aOR] = 1.44, 95% CI: 1.08–1.91), individuals with poor health literacy (aOR = 1.56, 95% CI: 1.31–1.87), and those residing in rural areas (aOR = 1.36, 95% CI: 1.18–1.58) had significantly greater odds of unmet healthcare needs. Among the need factors, those with fall experience (aOR = 1.56, 95% CI: 1.34–1.83), pain or discomfort (aOR = 2.40, 95% CI: 1.93–2.99), and high stress (aOR = 2.37, 95% CI: 2.02–2.79) had significantly higher odds of unmet healthcare needs (Table 3).

## 4. Discussion

We examined factors associated with unmet healthcare needs among older adults with osteoporosis in Korea using nationally representative KCHS data and Andersen’s behavioral model. Sex, health literacy, region, fall experience, pain/discomfort, and stress were all significantly associated with unmet healthcare needs. These results suggest that beyond healthcare accessibility, unmet healthcare needs among the older population with osteoporosis are associated with cognitive factors, physical symptoms, psychological burden, and disease-specific care requirements [11,12]. Because osteoporosis is closely associated with fragility fractures, disability, and long-term care needs, identifying these factors can help inform strategies to improve osteoporosis management and healthcare in this population [5,7,8,15].

Notably, females had higher odds of unmet healthcare needs than males did. This pattern is consistent with prior studies and may reflect differences in health status, healthcare utilization patterns, and social roles [19,21,29]. In osteoporosis, the higher prevalence among postmenopausal women may further increase symptom burden, fracture concerns, and long-term care needs [3,4,7,8]. However, we did not directly investigate interactions between sex and the other variables; as such, this association needs to be interpreted with caution. Future studies should examine whether sex modifies the relationships between health literacy, health status, and unmet healthcare needs.

Educational attainment was also associated with unmet healthcare needs in the univariable analysis, consistent with previous findings among older Korean adults [19,20]. However, this association was not maintained after adjustment, suggesting that more proximal cognitive factors such as health literacy, may partly explain the relationship between education and healthcare access. In osteoporosis care, formal education alone may not ensure that individuals understand the need for DXA screening, long-term medication adherence, exercise, calcium and vitamin D supplements, or fall-prevention strategies.

Health literacy emerged as another important factor associated with unmet healthcare needs. This association may be relevant to osteoporosis because the disease is often asymptomatic until fractures occur. Similar to formal education, limited health literacy may be associated with difficulties in understanding fracture risk, accessing DXA screening, interpreting treatment recommendations, or maintaining long-term osteoporosis management [9,29]. It may also be associated with challenges in communicating with healthcare providers and navigating follow-up care after falls, fractures, or worsening pain. Therefore, health education for these adults should use clear language, practical examples, visual materials, and repeated counseling where appropriate [30]. Such education should also cover fracture prevention, medication continuation, safe physical activity, nutrition, and when to seek medical care.

Among the need factors, pain/discomfort and stress were significantly associated with unmet healthcare needs. The high likelihood of unmet healthcare needs among individuals experiencing pain or discomfort is consistent with prior research involving patients with chronic conditions [12,14]. Patients with osteoporosis often experience persistent pain, functional decline, and reduced quality of life [8]. In osteoporosis, pain may be associated with vertebral compression fractures, musculoskeletal weakness, or functional impairment, These issues may, in turn, reduce mobility and make it difficult accessing healthcare services [5,7,8]. Consequently, these adults may experience greater barriers to healthcare utilization. Although we did not directly assess the reasons for delayed healthcare utilization, these barriers may partly explain why perceived healthcare needs do not always result in healthcare use [13].

Our findings highlight the importance of supportive strategies that facilitate appropriate healthcare access for older adults with osteoporosis. Community-based interventions, including home-based care and case management, can improve continuity of care and healthcare access for older adults with this chronic condition [13,31]. In osteoporosis care, these approaches should include the screening mentioned earlier, including fall-risk assessments, medication counseling, rehabilitation services, DXA screening, and follow-up programs [9,15].

Stress was also significantly associated with unmet healthcare needs, which is consistent with previous research reporting associations between mental health factors and unmet healthcare needs [12,19]. Psychological burdens, such as stress may be linked to delays or avoidance of healthcare utilization. This tendency may be more prevalent among older adults with chronic conditions, who manage both physical and psychological challenges. Among those with osteoporosis, stress may be intensified by chronic pain, fear of fractures, anxiety about falling, reduced mobility, and concerns about dependency. These psychological burdens may reduce the motivation to seek care, lower medication adherence, or decrease engagement in preventive behaviors. Thus, integrated approaches, including mental health support may be relevant in addressing unmet healthcare needs among patients with osteoporosis. Community-based interventions that provide ongoing counseling and emotional support could facilitate engagement with healthcare services [25].

Household income and economic activity were another significant determinants in the univariable analysis but not in the multivariable analysis, whereas region remained significant in both models. This finding diverges somewhat from prior research that identifies economic factors and healthcare accessibility as the primary determinants of unmet healthcare needs [11,22]. Our findings indicate that among older adults with osteoporosis, health status and health literacy were significantly associated with unmet healthcare needs, whereas economic factors were not significant in the multivariable model. One possible explanation is that the associations between enabling factors and unmet healthcare needs were weakened after adjusting for the need and cognitive factors in the model. Conversely, the association with region may reflect geographic differences in the availability of osteoporosis-specific resources, including DXA screening, specialist care, rehabilitation, and community-based fall-prevention programs.

Overall, our findings partially support Andersen’s behavioral model. While the predisposing and need factors were associated with unmet healthcare needs, consistent with previous studies [16,17], the comparatively limited role of the enabling factors suggests that patterns of healthcare utilization may differ in specific disease contexts. For conditions such as osteoporosis, where ongoing management and comprehension of health information are essential, cognitive factors and health status may be the most important factors associated with unmet healthcare needs. Osteoporosis management commonly requires patient-level understanding and sustained health behaviors, including adherence to anti-osteoporotic therapies, regular monitoring, exercise, nutrition, and fall-prevention practices. Furthermore, osteoporosis frequently coexists with frailty, sarcopenia, and functional decline in older adults, which may influence healthcare needs and utilization patterns beyond conventional socioeconomic enabling factors. Recent evidence has demonstrated a significant association between frailty and vertebral compression fractures among older adults with osteoporosis, with frail individuals exhibiting a higher prevalence of vertebral and multiple fractures. These findings suggest that frailty may represent an important underlying condition associated with greater healthcare needs and more complex care requirements among older adults with osteoporosis [32].

From a policy perspective, the results suggest that addressing unmet healthcare needs among patients with osteoporosis in Korea may require more than improving healthcare accessibility; namely, it may also warrant strengthening health literacy. Additionally, symptom-based management, including addressing pain and functional limitations, along with mental health support, are important considerations. Tailored education and continuous follow-up systems can help reduce disparities in healthcare utilization and promote more patient-centered care. More concrete osteoporosis-specific strategies could include improving access to DXA screening, supporting adherence to anti-osteoporotic therapies, providing fall-prevention and balance-training programs, expanding post-fracture care coordination, and integrating osteoporosis management with frailty and sarcopenia assessment in community and primary care settings.

However, several limitations of our study warrant mention in this discussion. First, because this was a cross-sectional study, causal relationships were not established. Second, all variables were measured using self-reported data, which may have been subject to recall and social desirability bias. Particularly, osteoporosis status was determined based on a self-reported physician diagnosis and may therefore be vulnerable to recall bias. Third, several variables, including health literacy, stress, and pain/discomfort, were assessed using single-item measures. Although these measures are practical in large-scale surveys, they may not fully capture the complexity of the constructs and can limit measurement reliability and validity. Fourth, key clinical characteristics, including disease severity, treatment status, and disease duration, were not fully examined. Specifically, osteoporosis severity, fracture history, vertebral compression fracture, medication adherence, use of anti-osteoporotic therapies, and prior DXA results were not available. Fifth, health literacy was assessed using a proxy indicator, which may not adequately reflect actual individual capacity. Sixth, other potentially relevant factors influencing healthcare utilization, such as psychological variables or the quality of healthcare services, were not considered. Residual confounding may also have remained because the dataset lack information on frailty, sarcopenia, depression, cognitive impairment, detailed mobility limitations, and specific type of health insurance coverage. Finally, differences between the univariable and multivariable findings may indicate the presence of interactions or confounding effects that were not fully accounted for in the final model.

Future studies could employ objective measures, such as DXA findings or KCD/ICD diagnostic codes, to improve osteoporosis assessments. They could also incorporate fracture history, treatment adherence, frailty or sarcopenia indicators, depression, and insurance-related variables to clarify the mechanisms underlying unmet healthcare needs in this population.

Despite these limitations, the study had several strengths. First, it utilized recent, nationally representative data, which enhanced the generalizability of our findings. Second, it offers a structured comprehensive analysis of unmet healthcare needs among adults with osteoporosis by applying Andersen’s behavioral model, demonstrating the utility and empirical application of this framework within disease-specific analyses. Future research could also employ longitudinal designs to clarify causal relationships and incorporate clinical variables alongside more precise measures of health literacy.

Ultimately, reducing unmet healthcare needs will require integrated approaches that address both individual and structural factors. For patients with osteoporosis, such approaches should be linked to a continuum of care, including early detection, timely treatment and long-term management.

## 5. Conclusions

We found that unmet healthcare needs among older adults with osteoporosis in Korea were significantly associated with sex, health literacy, region, fall experience, pain, and stress. These results imply that addressing unmet healthcare needs requires looking beyond healthcare accessibility. We must also consider an individuals’ ability to understand and use health information alongside their underlying physical and psychological health status. Therefore, interventions aimed at improving health literacy and addressing symptom-related factors, such as pain and stress, may help reduce unmet healthcare needs. Future strategies can incorporate both individual and structural approaches that promote improved healthcare among older adults with osteoporosis.

## Figures and Tables

**Table 1:** General characteristics of the older adults with osteoporosis (*n* = 20,988).

Variables	Categories	*N*	%
Predisposing factors			
Sex	Female	19,334	91.8
	Male	1654	8.2
Age	65–74	9866	52.5
	≥75	11,122	47.5
Educational attainment	≥High school	3953	28
	≤Middle school	17,026	72
Living with others	Yes	13,301	69.6
	No	7687	30.4
Health literacy	Good	8282	42.4
	Poor	12,681	57.6
Enabling factors			
Household income	High	7169	45.3
	Low	13,819	54.7
Economic activity	Employed	8612	30.2
	Unemployed	12,375	69.8
Region	Urban	8641	72.3
	Rural	12,347	27.7
Need factors			
Fall experience	No	16,054	75.4
	Yes	4934	24.6
Pain/Discomfort	No	6757	35.1
	Yes	14,231	64.9
Stress	Low	17,161	80.6
	High	3825	19.4

Values are presented as unweighted numbers (*n*) and weighted percentages (%). Sample sizes may vary owing to missing values.

**Table 2:** Comparison of general characteristics by unmet healthcare needs among older adults with osteoporosis (*n* = 20,988).

Variables	Categories	Unmet Needs		Rao–Scott *χ*^2^	*p*-Value
		No	Yes		
Predisposing factors					
Sex	Female	18,265 (91.7)	1069 (94.3)	8.70	0.003
	Male	1597 (8.3)	57 (5.7)		
Age	65–74	9418 (52.8)	448 (47.5)	8.14	0.004
	≥75	10,444 (47.2)	678 (52.5)		
Educational attainment	≥High school	3822 (28.3)	131 (20.9)	11.69	0.001
	≤Middle school	16,031 (71.7)	995 (79.1)		
Living with others	Yes	12,680 (69.8)	621 (64.2)	10.85	0.001
	No	7182 (30.2)	505 (35.8)		
Health literacy	Good	8001 (43.1)	281 (28.1)	60.56	<0.001
	Poor	11,837 (56.9)	844 (71.9)		
Enabling factors					
Household income	High	6916 (45.8)	253 (35.4)	25.43	<0.001
	Low	12,946 (54.2)	873 (64.6)		
Economic activity	Employed	8188 (30.2)	424 (30.8)	0.11	0.745
	Unemployed	11,673 (69.8)	702 (69.2)		
Region	Urban	8293 (72.7)	348 (63.6)	35.07	<0.001
	Rural	11,569 (27.3)	778 (36.4)		
Need factors					
Fall experience	No	15,322 (76.0)	732 (62.5)	71.75	<0.001
	Yes	4540 (24.0)	394 (37.5)		
Pain/Discomfort	No	6604 (36.1)	153 (15.2)	124.98	<0.001
	Yes	13,258 (63.9)	973 (84.8)		
Stress	Low	16,424 (81.6)	737 (60.8)	182.35	<0.001
	High	3436 (18.4)	389 (39.2)		

Values are presented as unweighted numbers (*n*) and weighted percentages (%). Differences between groups were measured using the Rao–Scott chi-square test. Statistical significance was set at *p* < 0.05.

**Table 3:** Factors associated with unmet healthcare needs among older adults with osteoporosis.

Variables	Categories	Crude OR (95% CI)	aOR (95% CI)
Predisposing factors			
Sex	Male (ref)	1.00	1.00
	Female	1.50 (1.14–1.95)	1.44 (1.08–1.91)
Age	65–74 (ref)	1.00	1.00
	≥75	1.24 (1.07–1.43)	0.92 (0.78–1.08)
Educational attainment	≥High school (ref)	1.00	1.00
	≤Middle school	1.49 (1.19–1.88)	0.97 (0.76–1.26)
Living with others	With others (ref)	1.00	1.00
	Living alone	1.29 (1.11–1.50)	1.10 (0.93–1.30)
Health literacy	Good (ref)	1.00	1.00
	Poor	1.94 (1.64–2.30)	1.56 (1.31–1.87)
Enabling factors			
Household income	High (ref)	1.00	1.00
	Low	1.54 (1.30–1.83)	1.15 (0.96–1.38)
Economic activity	Employed (ref)	1.00	1.00
	Unemployed	0.97 (0.83–1.14)	0.87 (0.74–1.02)
Region	Urban (ref)	1.00	1.00
	Rural	1.52 (1.32–1.75)	1.36 (1.18–1.58)
Need factors			
Fall experience	No (ref)	1.00	1.00
	Yes	1.90 (1.64–2.21)	1.56 (1.34–1.83)
Pain/Discomfort	No (ref)	1.00	1.00
	Yes	3.16 (2.56–3.91)	2.40 (1.93–2.99)
Stress	Low (ref)	1.00	1.00
	High	2.85 (2.43–3.33)	2.37 (2.02–2.79)

OR: Odds ratio; CI: Confidence interval; aOR: Adjusted odds ratio. Crude ORs were obtained using univariable logistic regression, and aORs were estimated using multivariable logistic regression. All the analyses accounted for the complex sampling design using stratification, clustering, and weighting. Statistical significance was set at *p* < 0.05.

## Data Availability

The data presented in this study are available in the Korea Community Health Survey repository at https://chs.kdca.go.kr/chs/rdr/rdrInfoProcessMain.do (accessed on 15 January 2026). These data were derived from the following resources available in the public domain: Korea Disease Control and Prevention Agency website.
